# Correlation between Density and Resorption of Fresh-Frozen and Autogenous Bone Grafts

**DOI:** 10.1155/2014/508328

**Published:** 2014-06-24

**Authors:** Simone Lumetti, Carlo Galli, Edoardo Manfredi, Ugo Consolo, Claudio Marchetti, Giulia Ghiacci, Andrea Toffoli, Mauro Bonanini, Attilio Salgarelli, Guido M. Macaluso

**Affiliations:** ^1^Università degli Studi di Parma, SbiBit, Via Gramsci 14, 43126 Parma, Italy; ^2^Università di Modena, Via Università 4, 41121 Modena, Italy; ^3^Università di Bologna, Via San Vitale 59, 40126 Bologna, Italy

## Abstract

*Trial Design*. This analysis compared the outcome of fresh-frozen versus autologous bone block grafts for horizontal ridge augmentation in patients with Cawood and Howell class IV atrophies. *Methods*. Seventeen patients received autologous grafts and 21 patients received fresh-frozen bone grafts. Patients underwent CT scans 1 week and 6 months after surgery for graft volume and density analysis. *Results*. Two autologous and 3 fresh-frozen grafts failed. Autologous and fresh-frozen grafts lost, respectively, 28% and 46% of their initial volume (*P* = 0.028). It is noteworthy that less dense fresh-frozen blocks lost more volume than denser grafts (61% versus 16%). *Conclusions*. According to these 6-month results, only denser fresh-frozen bone graft may be an acceptable alternative to autologous bone for horizontal ridge augmentation. Further studies are needed to investigate its behaviour at longer time points.

## 1. Introduction

Bone grafts are widely used to correct alveolar ridge atrophies in view of implant-supported rehabilitations. Autologous bone (AB) is currently considered the gold standard graft material for these procedures in spite of its significative drawbacks, as high morbidity due to the creation of a surgical donor site and limited availability, especially when harvested from intraoral sites [1–3]. Thus, alternative materials as fresh-frozen bone (FFB) from homologous donors have been proposed in recent years [4–9].

Bone grafts usually undergo extensive remodelling and resorption during the first year after surgery, which may affect the feasibility of an effective rehabilitation. A deeper understanding of those grafts' characteristics that can be predictive of their resorption is therefore of the utmost importance. Some authors suggested that graft resorption rate may be dependent on their embryologic origin, since grafts from membranous bone (as calvarial or mandibular grafts) do not resorb as extensively as those from endochondral bone (as iliac crest bone grafts) [10–12], although the reason for this phenomenon is still poorly understood. Other studies observed that cancellous bone grafts resorb faster than cortical bone grafts and thus concluded that resorption is mainly affected by graft structure and microarchitecture [13, 14]. Bone graft density may also be associated with their resorption, as it has been shown that high density grafts undergo a lower resorption than low-density grafts [15]. Relationship between bone resorption rate and graft density may represent an important parameter for understanding the mechanisms that regulate bone graft biological behaviour.

Bone density can be measured with high reproducibility by means of CT scans, which provide standardized values on the Hounsfield scale (HU). Other methods, which have been used, as Cone-Beam-Computed-Tomography (CBCT) or intraoral radiographies, do not guarantee appropriate accuracy in density determination [16]. CT scans can also be used to assess bone graft volume changes. Indeed, graft volume can be reconstructed based on CT data, as illustrated elsewhere [17, 18]. Other methods, such as linear measurements either with calipers and periodontal probes or on radiographies, do not provide tridimensional data of bone graft volume [19, 20].

This study aims to investigate whether a correlation between density and resorption of AB and FFB block grafts exists, by means of CT scans taken at 1 week (T0) and 6 months (T1) after grafting.

## 2. Materials and Methods

Thirty-eight patients (healthy, max 10 cigarettes/day) requiring one or multiple implants for partially or complete edentulism were enrolled in the study. Extensive written and verbal information was given to the patients before enrolment, and written informed consent was obtained. The study was approved by the Ethics Committee of Parma Province (Comitato Etico Unico di Parma).

Inclusion criteria were

- at least 18 years of age;
- Cawood and Howell class IV atrophy, defined as atrophic bone with knife edge alveolar ridge and inadequate width [4];
- adequate oral hygiene, that is, plaque index score and full mouth bleeding score ≤25%. Oral hygiene was improved until reasonable plaque and bleeding scores were obtained.

Exclusion criteria were

- previous radiotherapy to head and neck region;
- history of leucocyte dysfunction;
- history of bleeding disorders;
- history of renal failure;
- metabolic bone disorders;
- uncontrolled endocrine disorders;
- HIV infection;
- conditions requiring chronic use of antibiotics;
- use of steroids;
- alcohol or drug abuse;
- smoking >10 cigarettes a day (or cigar equivalents).

A locked computer software program (Minitab 1.5, Minitab, State College, PA, USA) was used to randomly allocate patients to receive AB or FFB block grafts. The allocation result was disclosed to the surgeon who was on the day of surgery. CT examiners were blinded to the allocation.

All patients received 2 g of amoxicillin 1 hour before surgery, as antibiotics prophylaxis. Immediately before surgery, all patients had a rinse with Chlorhexidine 0.2% for a minute.

AB blocks were harvested from intraoral sites (mandibular symphysis or retromolar trigone/mandibular ramus), while FFB blocks from tibial hemiplateau were provided by Banca del Tessuto Muscoloscheletrico (IOR, Bologna, Italy).

Before grafting surgery, FFB blocks were thawed in a 600 mg/L rifampicin and saline solution (Rifadin, Lepetit, Lainate, Italy) at 37°C, according to the provider's instructions. Then, after local anesthesia with articaine 4% and adrenaline 1 : 100.000 (Optocain, Molteni Dental S.p.A.), a trapezoidal mucoperiosteal flap was raised to allow the access to the recipient area. A midcrestal incision was made at mandibular sites, while at maxillary sites a beveled incision slightly palatal to the crest of the alveolar ridge was performed. The incision was continued in the gingival sulcus of the adjacent teeth when indicated. Buccal vertical releasing incisions were made to facilitate the surgical access and improve the mobility of the flap. The subperiosteal tissue was dissected to achieve an adequate visibility of the underlying bone. Then, the flap was gently elevated. The cortical bone of the recipient site was perforated with round or fissure burs under copious saline irrigation to create multiple communication with the marrow space, thus favoring the formation of the hematic clot and the blood supply from endosseous vessels. An incision through the periosteum at the base of the flap was made to allow the graft covering without any tension. The grafts were fixed in recipient sites with titanium screws (Cizeta Surgical, Bologna, Italy). Gaps around them were filled with bone chips. Collagen membranes (Osseoguard, Biomet 3i, Indiana, USA) were positioned on the grafts, as a covering. The closure of the wound for primary intention was obtained using monofilament sutures (Prolene 3-0 and 5-0, Ethicon, Johnson & Johnson, Amersfoort, The Netherlands). Antibiotics (amoxicillin, 2 g/day for 10 days) and pain medications were administered as needed.

All the patients underwent CT scans (Siemens CT4350) at T0 and T1. Computed tomographs were set as follows: gantry: 0, resolution: 512 × 512 pixel, WL (window level): 400, WW (window width): 4000, 130.00 Kv, 47 mA, exposure time: 800 ms, slice thickness: 1.25 mm, and slice reconstruction: 0.5 mm.

Acrylic radiographic templates were positioned to allow the realignment of different CT scans.

CT scans were analyzed as previously published [17]. Scan data were imported into a Dicom viewer software (OsiriX Imaging Software). Cross-sectional images perpendicular to the panoramic arch were constructed in the grafted area at an interval of 1 mm. The graft area was traced as a region of interest (ROI) freehand on the axial cross-sectional image. The grafts were tridimensionally reconstructed by computing all the selected 2d ROIs. Wherever graft margins were unclear, the grafted area was determined based on the morphology of the contralateral side. The total graft volume, its minimum, maximum, and mean density were obtained by stacking the calculated ROIs. Density was measured using the Hounsfield scale (HU).

Student's *t*-test was applied to evaluate differences in density change. Linear regression analysis and Pearson correlation test were used to investigate the correlation between parameters. The level of significance was set at *P* < 0.05.

## 3. Results

**Figure 1:**
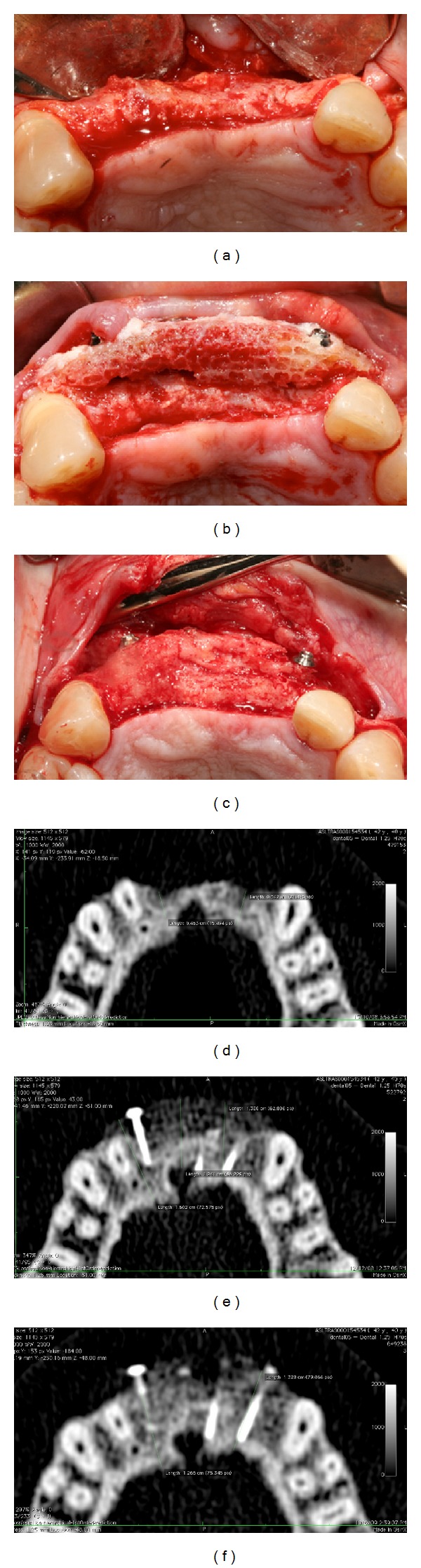
(a) Atrophic ridge before grafting; (b) homologous bone graft in place and (c) after 6 months of healing during surgery for implant placement. CT scans were taken before the intervention (d), 1 week after surgery (e), and after six months of healing (f).

Thirty-eight grafts were performed, 17 of AB and 21 of FFB (Figure 1). Thirteen AB and 13 FFB blocks were grafted in the maxilla, while 4 AB and 8 FFB blocks were grafted in the mandible.

Four graft exposures (1 maxillary AB, 1 maxillary FFB, 1 mandibular AB, and 1 mandibular FFB) occurred within the first 7 days after surgery. A further mandibular FFB completely resorbed by T2 and was considered a failure (Table 1). The grafts were surgically removed and the patients were excluded from further examinations. The characteristics of analyzed patients are summarised in Table 1.

**Figure 2:**
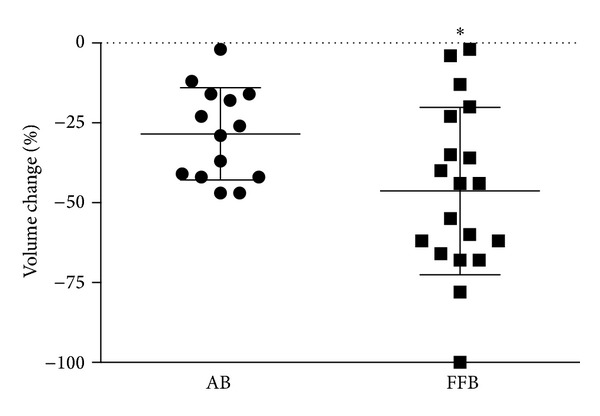
Graft depicting change in volume of AB and FFB grafts based on CT data after 6 months. The volume of grafts in both groups decreased over time, though to a greater extent for FFB grafts, *P = 0.028.

The initial volume of FFB and AB blocks was not significantly different (1.22 ± 0.86 cm^3^ versus 0.74 ± 0.98 cm^3^, *P* = 0.15). At T2 both AB and FFB grafts underwent extensive remodelling as evidenced by volume change at CT scans, but FFB showed significantly more resorption. AB lost an average of 28% of the initial volume, whereas FFB decreased by 46% (*P* = 0.028) (Figure 2). Interestingly, in one case an FFB graft was completely resorbed and could not be observed at the second CT scan.

**Figure 3:**
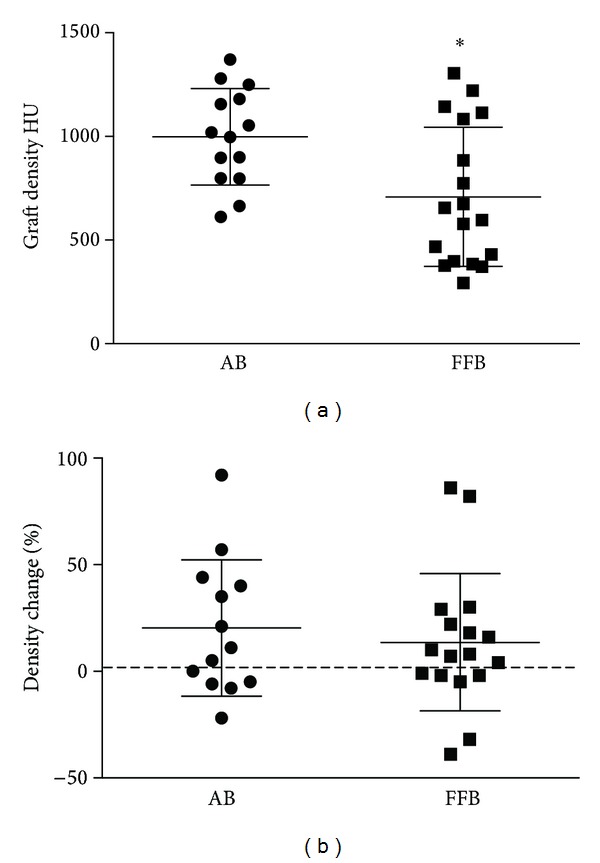
Graft density as determined 1 week after insertion at CT (a) and graft density change after 6 months of healing (b). Density of AB grafts was significantly higher than FFB grafts, *P = 0.099.

**Figure 4:**
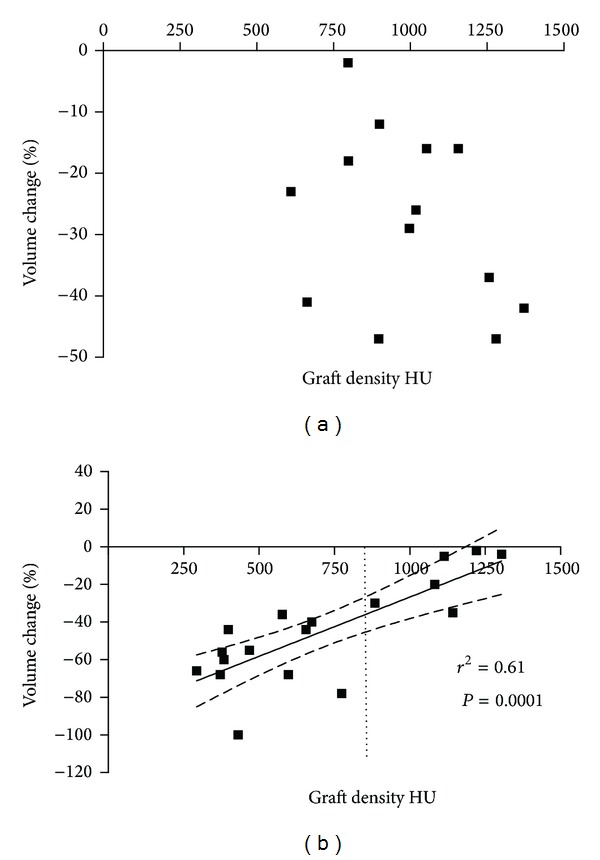
Correlation between graft density and volume change over 6 months for AB (a) and FFB (b) grafts. No correlation was found for AB, but a linear relation between these parameters existed for FFB (*r* ^2^ = 0.61, *P* = 0.0001).

The mean initial density of homologous bone grafts was 708 ± 335 HU and it was significantly lower (*P* = 0.0099) than the density of autologous bone grafts (998 ± 232 HU) (Figure 3(a)). The mean density variation was 20.31% in the control group and 13.59% in the test group (Figure 3(b)). The difference between the groups was not statistically significant (*P* = 0.52). The Pearson test revealed that no correlation between initial density and degree of resorption existed for autologous bone grafts (Figure 4(a)), while such a correlation was significant for fresh-frozen bone grafts (Figure 4(b)). Less dense grafts tended to lose more volume than denser grafts: average volume resorption for <800 HU (Figure 4(b), dashed line) fresh-frozen bone was −57%, whereas it was −15% when initial density was >800 HU (*P* = 0.001). Surgery did not affect AB and FFB graft resorption.

## 4. Discussion

To the best of our knowledge, no studies about the correlation between volume and density of fresh-frozen homologous bone are available in scientific literature. We believe that a more detailed awareness of fresh-frozen bone graft changes over time would be useful for clinicians in order to ameliorate their daily practice.

In our analysis, CT scans revealed that both AB and FFB grafts underwent extensive resorption at 6 months, and FFB grafts lost significantly more volume. FFB resorption showed, however, high variability, with wide differences from case to case, ranging from complete resorption to almost no change in graft volume. These results are in line with those presented in several other studies [21–23] and show a poor predictability of graft volume resorption.

Based on our results, it was possible to highlight a linear correlation between initial density of FFB grafts and their resorption at T1, as denser grafts showed less resorption than low-density grafts. Such a correlation was independent of graft embryologic origin, as all FFB blocks of this study were harvested from tibia. FFB grafts had a wide density range, depending on the portion of tibia they were harvested from. Indeed, tibia is a long bone that possesses a large epiphysis that tapers down into a narrower, denser diaphysis, mainly composed of thick cortical bone with high HU values. On the other hand, AB grafts harvested from intraoral sites (either mandibular symphysis or ramus) had a limited density range and this may have hampered the possibility to find a correlation between density and resorption, which has been observed in clinical and preclinical reports [14, 24]. It is however noticeable that density of AB grafts was comparable to that of denser FFB grafts and thus was their resorption.

Previous studies have shown a correlation between bone density and their structure and go as far as possible to propose a density-based classification for bone quality [25]. Thus, it is supposable that in this case FFB graft architecture, in terms of cortical and cancellous composition, may have played a role in their resorption. Spin-Neto reported that cortical FFB grafts were not significantly remodelled after 5 months. Neither newly formed bone nor pristine bone was in contact with the graft, which showed necrotic portions, osteoclastic activity, and areas invaded by dense connective tissue [26]. On the contrary, Orsini observed that corticocancellous grafts were well integrated in the recipient areas, grafted bone was in close continuity with new bone, and marrow spaces contained small newly formed vessels [27]. These considerations highlight that cortical grafts are hard and resistant to vascular penetration but they are progressively weakened by degeneration before their complete incorporation and remain as admixtures of necrotic and viable bone for prolonged periods of time. On the contrary, cancellous grafts are remodelled and revascularized more rapidly than cortical grafts but they generally undergo a greater resorption. FFB grafts remodelling and their clinical relevance have however still to be investigated.

It has also to be noted that other variables, like donor's age and sex, may affect bone graft performances; nevertheless no data are available to this regard.

Five grafts failed shortly after placement. However, these failures were quite evenly distributed across the groups, as both autologous and homologous grafts failed regardless of their graft site. It is pure speculation to fathom why these failures occurred, given that the no noticeable deviation from surgical protocol occurred with these patients. Failures cannot be attributed to graft type or to graft characteristics, such as density or volume, as these did not significantly differ from successful grafts.

Based on our findings, we may conclude that FFB grafts with a density >800 HU are clinically preferable to less dense grafts, due to their lower degree of resorption. Further studies that analyse the behaviour of AB grafts with a wider density range are recommended.

## Figures and Tables

**Table 1:** This summarises age, gender, and edentulous site of treated patients.

Patient	Age	Gender	Edentulous site	Notes
1	46	M	Anterior maxilla	
2	56	F	Posterior mandible	
3	22	F	Posterior mandible	
4	24	M	Anterior mandible	
5	56	F	Posterior maxilla	
6	54	F	Posterior mandible	
7	49	F	Posterior maxilla	
8	30	M	Anterior maxilla	
9	43	M	Anterior maxilla	
10	54	F	Posterior maxilla	
11	51	F	Posterior maxilla	
12	53	M	Posterior maxilla∗	Failed (complete resorption)
13	60	M	Posterior maxilla	
14	52	F	Posterior maxilla	
15	55	F	Posterior maxilla	
16	55	F	Posterior maxilla	
17	45	F	Posterior maxilla	
18	61	F	Posterior maxilla	
19	61	F	Posterior maxilla	
20	61	M	Posterior maxilla	
21	53	F	Posterior maxilla	
22	53	F	Posterior maxilla	
23	52	M	Posterior mandible	
24	51	M	Posterior maxilla	
25	74	F	Posterior mandible	
26	52	M	Posterior mandible	
27	55	F	Posterior maxilla	
28	70	F	Posterior mandible	
29	53	F	Anterior mandible∗	Failed (graft exposure)
30	41	M	Anterior maxilla∗	Failed (graft exposure)
31	76	F	Anterior maxilla	
32	64	F	Anterior maxilla	
33	37	F	Anterior maxilla	
34	45	M	Anterior maxilla	
35	61	F	Anterior maxilla	
36	55	F	Posterior maxilla	
37	64	M	Posterior maxilla∗	Failed (graft exposure)
38	53	F	Posterior mandible∗	Failed (graft exposure)

Failures are marked with an asterisk.
